# Exploring the path to polio eradication: insights from consecutive seroprevalence surveys among Pakistani children

**DOI:** 10.3389/fpubh.2024.1384410

**Published:** 2024-03-27

**Authors:** Imtiaz Hussain, Muhammad Umer, Ahmad Khan, Muhammad Sajid, Imran Ahmed, Kehkashan Begum, Junaid Iqbal, Muhammad M. Alam, Rana M. Safdar, Shahzad Baig, Arie Voorman, Jeffrey Partridge, Sajid Soofi

**Affiliations:** ^1^Center of Excellence in Women and Child Health, The Aga Khan University, Karachi, Pakistan; ^2^Department of Pediatrics and Child Health, The Aga Khan University, Karachi, Pakistan; ^3^National Institute of Health (Pakistan), Islamabad, Pakistan; ^4^Polio National Emergency Operations Center, Islamabad, Pakistan; ^5^Bill and Melinda Gates Foundation, Seattle, WA, United States

**Keywords:** poliovirus, type 2 immunity, eradication, seroprevalence, Pakistan

## Abstract

**Introduction:**

After trivalent oral poliovirus vaccine (tOPV) cessation, Pakistan has maintained immunity to type 2 poliovirus by administering inactivated polio vaccine (IPV) in routine immunization, alongside monovalent OPV type 2 (mOPV2) and IPV in supplementary immunization activities (SIAs). This study assesses the change in poliovirus type 2 immunity after tOPV withdrawal and due to SIAs with mOPV2 and IPV among children aged 6–11 months.

**Methods:**

Three cross-sectional sequential serological surveys were conducted in 12 polio high-risk areas of Pakistan. 25 clusters from each geographical stratum were selected utilizing probability proportional to size.

**Results:**

Seroprevalence of type 2 poliovirus was 49%, with significant variation observed among surveyed areas; <30% in Pishin, >80% in Killa Abdullah, Mardan & Swabi, and Rawalpindi. SIAs with IPV improved immunity from 38 to 57% in Karachi and 60 to 88% in Khyber. SIAs with IPV following mOPV2 improved immunity from 62 to 65% in Killa Abdullah, and combined mOPV2 and IPV SIAs in Pishin improved immunity from 28 to 89%. Results also reflected that immunity rates for serotypes 1 and 3 were consistently above 90% during all three phases and across all geographical areas.

**Conclusion:**

The study findings highlight the importance of implementing effective vaccination strategies to prevent the re-emergence of poliovirus. Moreover, the results provide crucial information for policymakers working toward achieving global polio eradication.

## Introduction

Globally, progress has been made toward eradicating wild poliovirus (WPV), with two of the three strains; (WPV2 and WPV3) successfully eliminated (1). Nonetheless, the transmission of wild poliovirus type one (WPV1) continues in two neighboring countries of Pakistan and Afghanistan (2). Although Pakistan has made significant progress in reducing the prevalence of poliovirus in recent years, the transmission of wild poliovirus is not yet fully contained. Despite the country reporting an all-time low number of WPV 1 cases, 8 in 2017 and 12 in 2018, the number of cases surged to 147 in 2019, and 84 in 2020. However, in 2021, only 1 case of WPV1 was reported followed by a rise to 20 in 2022 and a reduction to 06 cases in 2023 (3, 4). Additionally, the suspension of polio campaigns for five months in Pakistan during the COVID-19 pandemic in 2020 left 40 million children without their polio vaccine (5). Moreover, despite continued efforts and progress over the past years, routine immunization coverage in Pakistan also remains suboptimal at 76.5%, with polio vaccines coverage of 84.2% for both; the inactivated poliovirus vaccine (IPV) and the third dose of the oral polio vaccine (OPV3) (6).

The oral poliovirus vaccine (OPV), which comprises live-attenuated strains of poliovirus 1, 2, and 3 has been instrumental in eradicating polio globally (7–9). However, a major drawback of OPV is its potential to cause paralytic diseases in rare cases (10). OPV has the potential to induce vaccine-associated paralytic poliomyelitis (VAPP) in individuals who receive the vaccine and in those in close proximity. VAPP occurs when attenuated oral poliovirus vaccine relapses sometimes to neurovirulence, leading to paralysis that clinically resembles poliomyelitis caused by wild poliovirus (WPV) (7, 10). Furthermore, OPV viruses excreted by the vaccine recipients and their close contacts may regain their transmissibility and neurovirulence after losing their attenuating mutations. This could result in outbreaks of vaccine-derived poliovirus (VDPV) poliomyelitis akin to those caused by wild polioviruses (10, 11). The type 2 virus of the trivalent oral poliovirus vaccine (tOPV) accounted for more than 95% of cVDPV outbreaks and around 30% cases of VAPP between 2006 and 2016 (10, 11). Therefore its use has globally ceased since April 2016 (12, 13). The change from tOPV to bivalent OPV (bOPV) was introduced to alleviate the burden of VAPP cases. But Sabin-like type 2, which was circulating before the withdrawal of tOPV, could continue to cause outbreaks of type 2 cVDPV (12, 14, 15). In 2019, Pakistan reported 22 cases of cVDPV. However, during the pandemic in 2020, these cases increased to 135, warranting an OPV2 response in the country (4). To counteract type 2 cVDPV, monovalent OPV2 (mOPV2) was used with bOPV alongside inactivated polio vaccine (IPV) in routine immunization and supplementary immunization activities (SIA) (9, 16, 17). Though IPV does provide mucosal immunity but not as effective as OPV, and it can enhance mucosal immunity in individuals who have received OPV previously. Furthermore, IPV can also restrict pharyngeal shedding, a minor contributor to transmission and does not markedly decrease the prevalence of poliovirus in stool samples (16, 18). Therefore, mOPV2 vaccination campaigns in target areas are recommended to contain the circulation of cVDPV (19).

This study presents the results of a series of three seroprevalence surveys conducted in 12 polio high-risk areas in Pakistan, targeting children between 6 to 11 month of age, to understand the change in PV2 immunity after tOPV cessation, and assesses the effect of mOPV2 and IPV on population seroprotection against type 2 poliovirus. Moreover, the study has also compared the immunity of children before and after of SIAs containing mOPV2 and multiple IPV campaigns that were conducted in the country, responding to the poliovirus outbreak. The findings from the first and second rounds of the survey have already been reported in another study (9).

## Materials and methods

### Survey methodology

A series of cross-sectional serological surveys were conducted between November 2016 and April 2019 by the Aga Khan University (AKU) Karachi in close coordination with the national and provincial emergency operations centers for polio eradication. The surveys were conducted in three phases, and in each phase, it was conducted in 12 geographic strata, which were identified as the polio high risk areas by the National Emergency Operations Cell (NEOC). These include Karachi, Larkana, and Sukkur from Sindh, Quetta, Killa Abdullah, and Pishin from Balochistan, Peshawar, and Mardan & Swabi from Khyber Pakhtunkhwa (KP), and Khyber from erstwhile Federally Administered Tribal Areas (FATA) in KP now as Khyber Pakhtunkhwa-Newly Merged Districts (KP-NMD). Data were also collected from three control sites one in each phase, i.e., Rawalpindi (in phase 1), Lahore (in phase 2), and Multan (in phase 3). As a reference point and a comparator to the high-risk areas, the control sites were selected as area of low risk.

For each phase of the survey, 25 clusters were chosen from each geographical stratum employing probability proportional to size (PPS) and the lot quality assurance sampling (LQAS) sampling frame of the polio program. The vaccination areas being assigned by the polio program were considered as clusters. These clusters serve as the primary sampling unit for the study. Household listing was conducted after identifying the clusters, and twelve households with a child between the age of 6 to 11 months were randomly selected. From each household, only one target child was selected, and if a household had more than one child of the target age group, the Kish grid method was used to select one child. To estimate seroprevalence with a 95% confidence margin of ± 5% assuming true Seroprevalence 85%, design effect of 1.5 and 90% individual response rate, the required sample size was 294 per stratum, and we covered a sample of 300 from each strata. During surveys, households that were locked or those who refused to participate were not replaced with other households. Information on demographics and socio-economic status and history on vaccination was obtained for all children included in the surveys using a structured questionnaire designed for administration to the to mothers or caregivers of the targeted children. The questionnaire underwent translation into the national language (Urdu) and pilot field-testing on 100 samples across five different locations. Trained phlebotomists also took 2 mL of venous blood from each child.

The survey team was composed of data collectors, phlebotomists, lab technicians, social mobilizers, team leaders, medical officers, and regional supervisors. Each data collection team included two female data collectors, two female phlebotomists, one male and one female social mobilizer, and a male team leader. Before initiating field activities, team leaders and supervisors underwent comprehensive central training in Karachi. Conducted over five days, this training program was led by experienced investigators and faculty members from Aga Khan University. Subsequent cascading training sessions were held for the data collection teams at the regional level. Ethical approval was obtained from the Ethical Review Committee of Aga Khan University, Pakistan, and the National Bioethics Committee, Pakistan. Informed consent was obtained from the caregivers of the participants included in the study.

### Laboratory methodology

Serum was isolated and put into sterile, labeled cryovials following centrifugation. The cryovials were kept in a cold box with ice packs and sent straight away to the nearest laboratory collection point of AKU, and then transported to the Nutrition Research Laboratory at AKU in Karachi. A digital thermometer fixed to the lid of the ice box was used to monitor its temperature during transportation to the collection point and subsequently during onward transportation to AKU Karachi. Two distinct aliquots were made in the laboratory; one was kept as a backup and the other was sent to the Centers for Disease Control in Atlanta, USA, for neutralization assay examination (20). For this study, seropositivity was defined as the titer of poliovirus neutralizing antibody >1:8 (21). AKU has a strong network of laboratories that spans across more than 290 locations throughout Pakistan, covering over 100 cities. These clinical Laboratories are accredited by the College of American Pathologists (CAP) and we used this network for transportation of the study samples.

### Statistical methodology

The primary outcome of this study was the prevalence of type 2 seropositivity. Overall seropositivity was analyzed separately by geography, survey rounds, serotype, and birth cohort of the children. Survey data were supplemented with information on the number of SIAs that each child would have been eligible for, and overall seropositivity rates were calculated accordingly. Pakistan followed the golobal synchronization and discontinued the tOPV on May 12, 2016. IPV was included in the routine immunization schedule of Pakistan on August 24, 2015, and incorporated into SIAs from November 2015 (12, 13, 22). During SIAs, children less than 5 years of age were targeted for bOPV and children between 4 and 23 months of age were targeted for IPV. Therefore, children under the age of 4 months were excluded from analyses of IPV if they were included in SIAs for IPV. Immunity was only evaluated for children who were born after the withdrawal of tOPV (after May 2016) i.e. those who were ineligible for SIAs including type 2 vaccine.

To determine the impact of SIAs, we calculated immunity by area and eligibility of children for SIA. We excluded the immune response to vaccines administered during SIAs within 14 days of the blood draw from our analysis. Additionally, we estimated the intention to treat (ITT) seroconversion wherever possible. We assigned similar weights to different strata to ensure that the assessment of immunity represents all areas fairly without being influenced by areas with high-population averages like Karachi. For the analyses, three strata of Karachi and two strata of Peshawar were combined into a separate single unit; however, three strata from Quetta (Quetta, Killa Abdullah, and Pishin), two from KP-NMD and KP (Khyber, and Mardan & Swabi), and two from Northern Sindh (Sukkur, and Larkana) were separately reported. R software was used for analyses (23).

## Results

### Sample population

**Figure 1:**
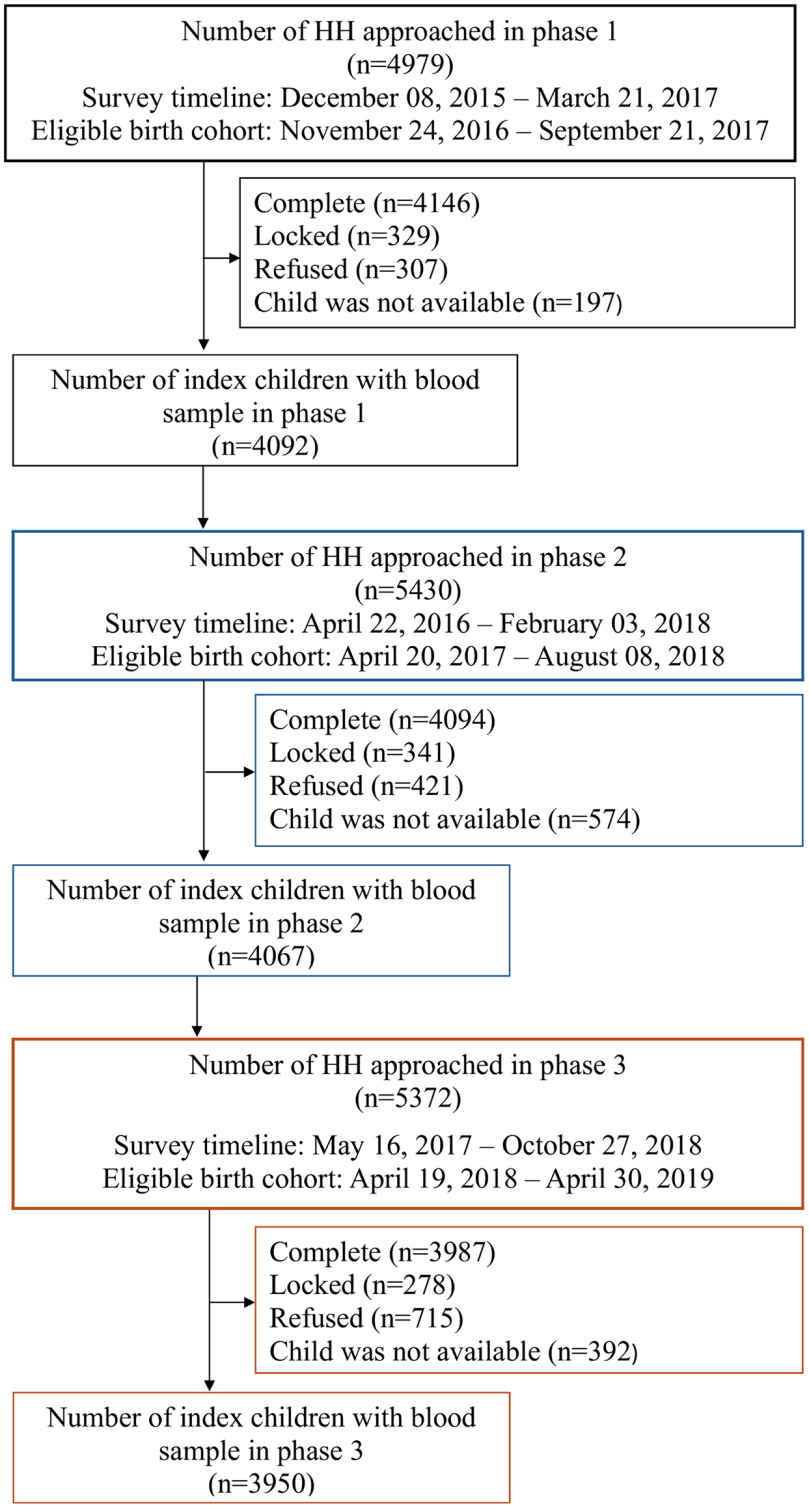
Study profile.

The study approached a total number of 15,781 households from the three phases of the survey, with 4,979 from Phase one, 5,430 from phase two and 5,372 from phase three. Altogether 12,227 children under five years of age were covered, and blood samples were drawn for 12,109 children. Figure 1 presents the coverage of households, eligible children, their birth cohort and survey timeline for each phase of the survey.

Table 1 details the demographic characteristics of 12,227 children from 975 primary sampling units covered during the three phases of the survey from the selected areas. The illiteracy rate among respondents ranged from 19% in Rawalpindi to 100% in district Killa Abdullah, and the preponderance of male children varied from 46 to 63% in Larkana and Khyber, respectively. Immunization cards were available with more than 50% of the children in all target areas except Killa Abdullah, Pishin, Larkana, Sukkur, and Karachi. Furthermore, OPV3 coverage was lowest in Killa Abdullah (21%) and highest in Multan (100%). Similarly, the IPV coverage was lowest in Killa Abdullah (26%) and highest in Khyber (100%).

**Table 1:** Basic demographic characteristics of study areas.

Area	Survey phase	N	Clusters	Education level (Illiteracy rate- %)	Male Children (%)	Vaccination card (%)	OPV3 coverage (%)	IPV coverage (%)
Karachi	1	1,003	75	38.8	52	73	73	79
	2	971	75	33.7	53.3	68.5	63.5	74.4
	3	936	75	53.1	50.6	46	65.7	78.7
Quetta	1	311	25	83.9	49.8	65.6	95.5	62.7
	2	317	25	91.5	51.1	49.8	54.3	60.6
	3	310	25	72.3	61.9	60.6	31.6	75.2
Killa Abdullah	1	312	26	98.7	50.3	34.3	55.1	26
	2	326	25	100	51.5	40.2	33.7	39.9
	3	314	25	84.1	53.5	28.3	20.7	48.1
Pishin	1	309	24	89.6	47.2	56.3	91.6	59.5
	2	310	25	96.5	50.3	49.7	44.5	50
	3	304	25	92.8	54.6	48.4	33.2	58.9
Peshawar	1	627	50	80.9	53.1	84.5	76.7	83.1
	2	651	50	82.6	49.2	91.1	83.7	89.1
	3	619	51	65.9	48.3	91.3	89.3	91.6
Khyber	1	302	25	95.4	63.2	72.5	72.8	62.9
	2	300	25	94	59.7	69	99	100
	3	302	23	88.4	49.7	90.4	98.3	100
Mardan & Swabi	1	314	25	63.1	56.1	85.7	89.8	93.9
	2	309	25	68	51.1	57	88.7	86.4
	3	298	25	32.6	52.7	99	91.3	98.3
Larkana	1	341	25	88.6	46	43.4	58.4	66.3
	2	313	25	80.2	48.2	54	90.4	86.9
	3	303	25	84.8	48.8	84.2	88.4	90.4
Sukkur	1	300	25	75	50.7	44.7	44.3	61.7
	2	298	25	94.3	53.7	33.6	35.9	55.7
	3	300	25	92	54.7	72.3	69.3	78
Control sites								
Rawalpindi	1	327	26	19.3	51.7	93	95.4	97.6
Lahore	2	299	25	25.8	54.2	90.3	96	97
Multan	3	301	25	66.4	55.1	87.4	99.7	99.3

N, sample size; IPV, inactivated polio vaccine; OPV3, third dose of oral polio vaccine.

Table 2 displays the timings of the survey, blood drawn, birth cohort and eligibility of children for IPV and mOPV2 SIA in each target area in each survey phase. Data and sample collection were completed between November 2016 and April 2019 with a median collection time of 24 days (range 8–151 days) for a single phase in a certain geographic area. Children surveyed were born between December 2015 and October 2018. The majority of the children surveyed in the first phase were born prior to the change from tOPV to bOPV with the percentage ranging from 27% in Rawalpindi to 62% in Peshawar. During the second phase of the survey, more than 70% of the children from Quetta, Killa Abdullah, Pishin, Khyber, Peshawar, and Sukkur were eligible for IPV SIA, whereas only children from Quetta, Killa Abdullah, Pishin, and Peshawar remained eligible during the third phase. In addition to that, children from Pishin, Quetta, and Killa Abdullah were eligible for mOPV2 SIA during the second phase, while none were eligible during the third phase of the survey.

**Table 2:** Survey timing, birth cohort, and eligibility for IPV and mOPV2 SIAs in each study area.

Area	Survey phase	Blood drawn		Birth cohorts		Born before switch (%)	IPV SIA eligibility (%)	mOPV2 SIA eligibility (%)
Karachi	1	16-Nov	17-Mar	15-Dec	16-Sep	50.6	35	0
	2	17-Apr	17-Sep	16-May	17-Mar	0	49.3	0
	3	18-Apr	18-Aug	17-May	18-Mar	0	0	0
Quetta	1	17-Jan	17-Feb	16-Feb	16-Aug	40.2	0	97.7
	2	17-Jul	17-Jul	16-Aug	17-Jan	0	81.4	100
	3	18-Sep	18-Oct	17-Oct	18-Apr	0	100	0
Killa Abdullah	1	17-Mar	17-Mar	16-Apr	16-Sep	3.8	0	100
	2	17-Aug	17-Aug	16-Sep	17-Feb	0	72.1	95.4
	3	18-Oct	18-Oct	17-Nov	18-May	0	44.3	0
Pishin	1	16-Dec	17-Jan	16-Jan	16-Jul	52.1	0	1
	2	17-May	17-Jul	16-Jun	17-Jan	0	94.2	100
	3	18-Sep	18-Oct	17-Oct	18-Apr	0	100	0
Peshawar	1	16-Dec	17-Feb	15-Dec	16-Aug	61.9	8.9	0
	2	17-Apr	17-Jul	16-Apr	17-Jan	0	70.7	0
	3	18-Apr	18-Sep	17-Jun	18-Mar	0	76.4	0
Khyber	1	17-Sep	17-Sep	16-Sep	17-Mar	0	17.2	0
	2	18-Jul	18-Aug	17-Aug	18-Feb	0	89	0
	3	19-Apr	19-Apr	18-Apr	18-Oct	0	0	0
Mardan & Swabi	1	17-May	17-May	16-May	16-Nov	0	76.1	0
	2	17-Oct	17-Oct	16-Oct	17-Apr	0	0	0
	3	18-Aug	18-Sep	17-Sep	18-Mar	0	0	0
Larkana	1	17-Jan	17-Feb	16-Jan	16-Aug	47.8	0	0
	2	17-Aug	17-Aug	16-Aug	17-Feb	0	32.3	0
	3	18-Apr	18-May	17-May	17-Nov	0	0	0
Sukkur	1	16-Dec	17-Jan	15-Dec	16-Jul	53.3	0	0
	2	17-Apr	17-May	16-May	16-Nov	0	74.8	0
	3	18-Apr	18-May	17-May	17-Nov	0	0	0
Control sites								
Rawalpindi	1	17-Mar	17-Mar	16-Mar	16-Sep	27.2	0	0
Lahore	2	17-May	17-May	16-Jun	16-Dec	0	0	0
Multan	3	18-Nov	18-Dec	17-Dec	18-Jun	0	0	0

IPV, inactivated polio vaccine; SIA, supplementary immunization activities; mOPV2, monovalent oral poliovirus type 2 vaccine.

### Overall seroprevalence

**Figure 2:**
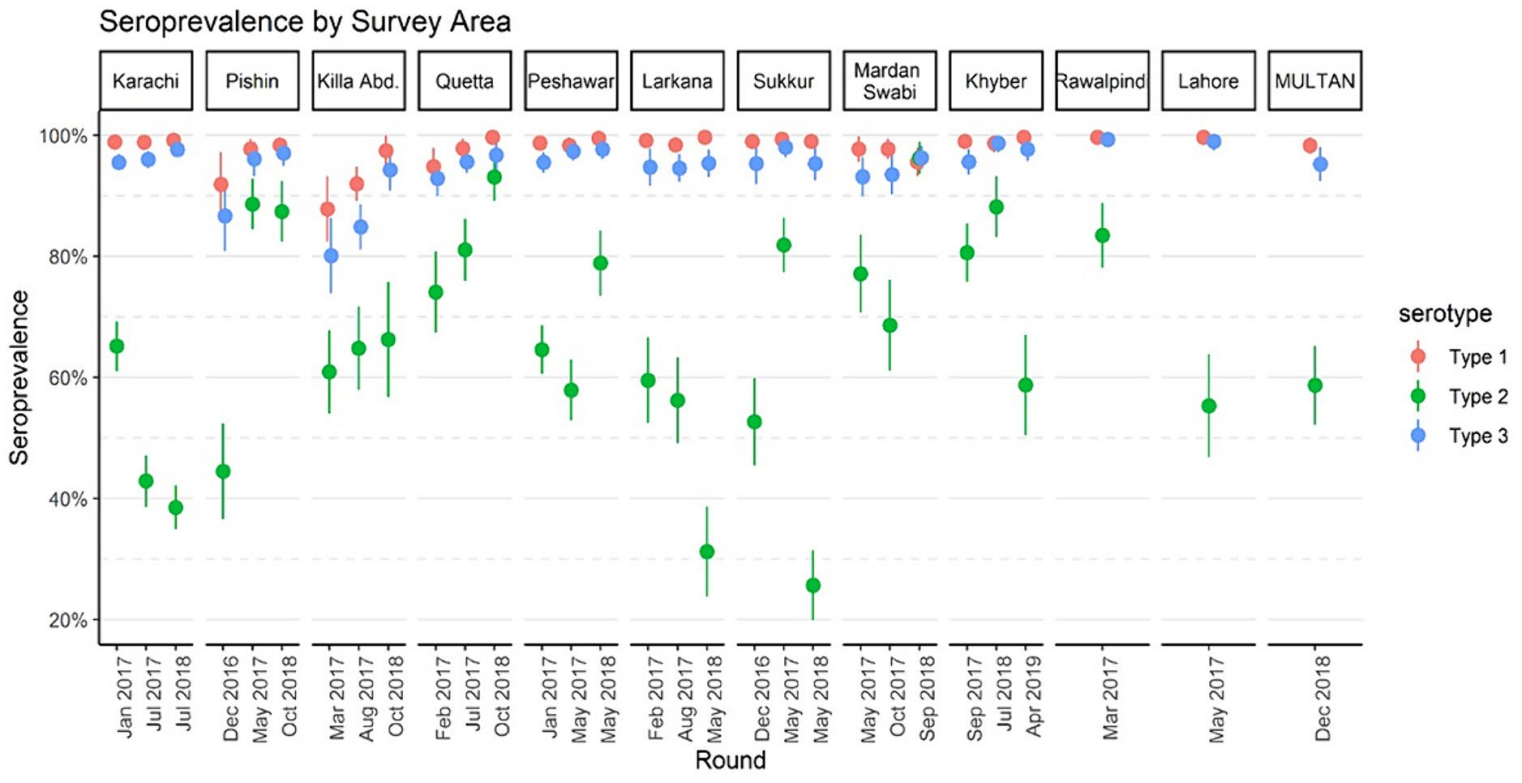
Seroprevalence by survey areas of Pakistan, 2016–2019. The points denote seroprevalence estimates, and lines indicate 95% confidence intervals. The *x*-axis represents the median month of the blood draw for children in the survey round.

**Figure 3:**
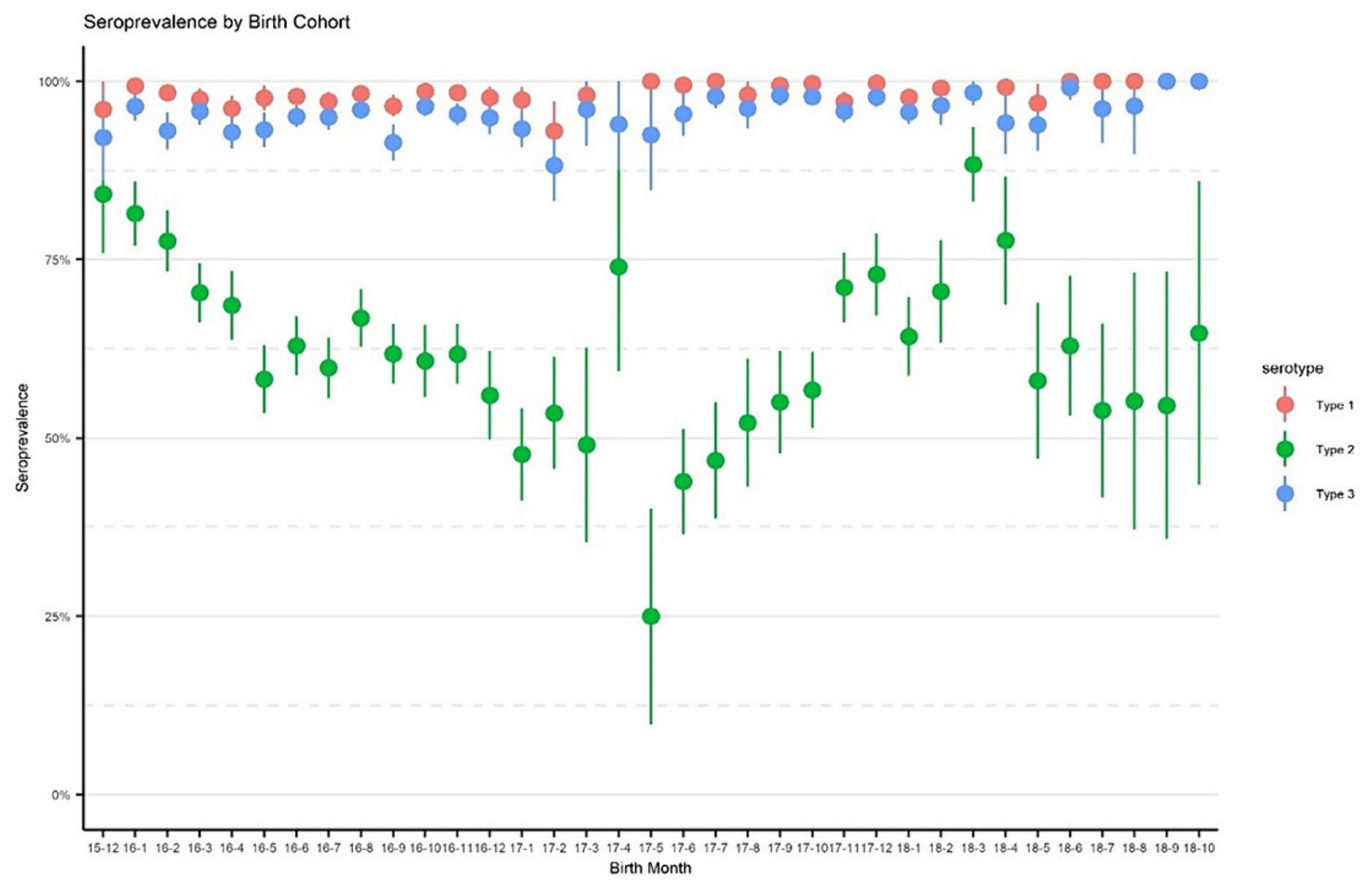
Seroprevalence by birth cohorts. The points denote seroprevalence estimates, and lines indicate 95% confidence intervals. The *x*-axis represents birth in year-month format.

Figure 2 displays immunity rates of serotypes 1, 2, and 3 by survey areas in each phase of the survey. Immunity rates for serotypes 1 and 3 were consistently above 90% during all three phases and across all geographical areas including the control sites, with a few exceptions for Pishin and Killa Abdullah. Serotype 3 was lower than 90% during the first phase in Pishin and was lower in the first and second phase in Killa Abdullah. Also, serotype 1 was below 90% in Killa Abdullah in the first phase. However, the immunity for type 2 varied across the survey areas, ranging from 26% in Sukkur in May 2018 to 96% in Mardan & Swabi in September 2018. In the control sites, the immunity for type 2 was similar to the intervention areas with 57% in Lahore, 59% in Multan and 81% in Rawalpindi. The seroprevalence by birth cohort is presented in Figure 3. The results indicated that immunity was consistently higher for type 1 and type 3 across all birth cohorts, while for type 2, it varied significantly. Specifically, children born in 2015 were eligible for tOPV vaccination in routine immunization and SIAs conducted before the switch to bOPV, their immunity was recorded over 75%. After the switch to bOPV, the immunity rate dropped to 25%. However, in 2019, when cVDPV cases surfaced in the country, mOPV2 was administered during SIAs and it resulted in a significant improvement in seroprevalence for type 2 poliovirus with immunity rates exceeding 50% among the target age group.

### Effectiveness of IPV through routine immunization

**Figure 4:**
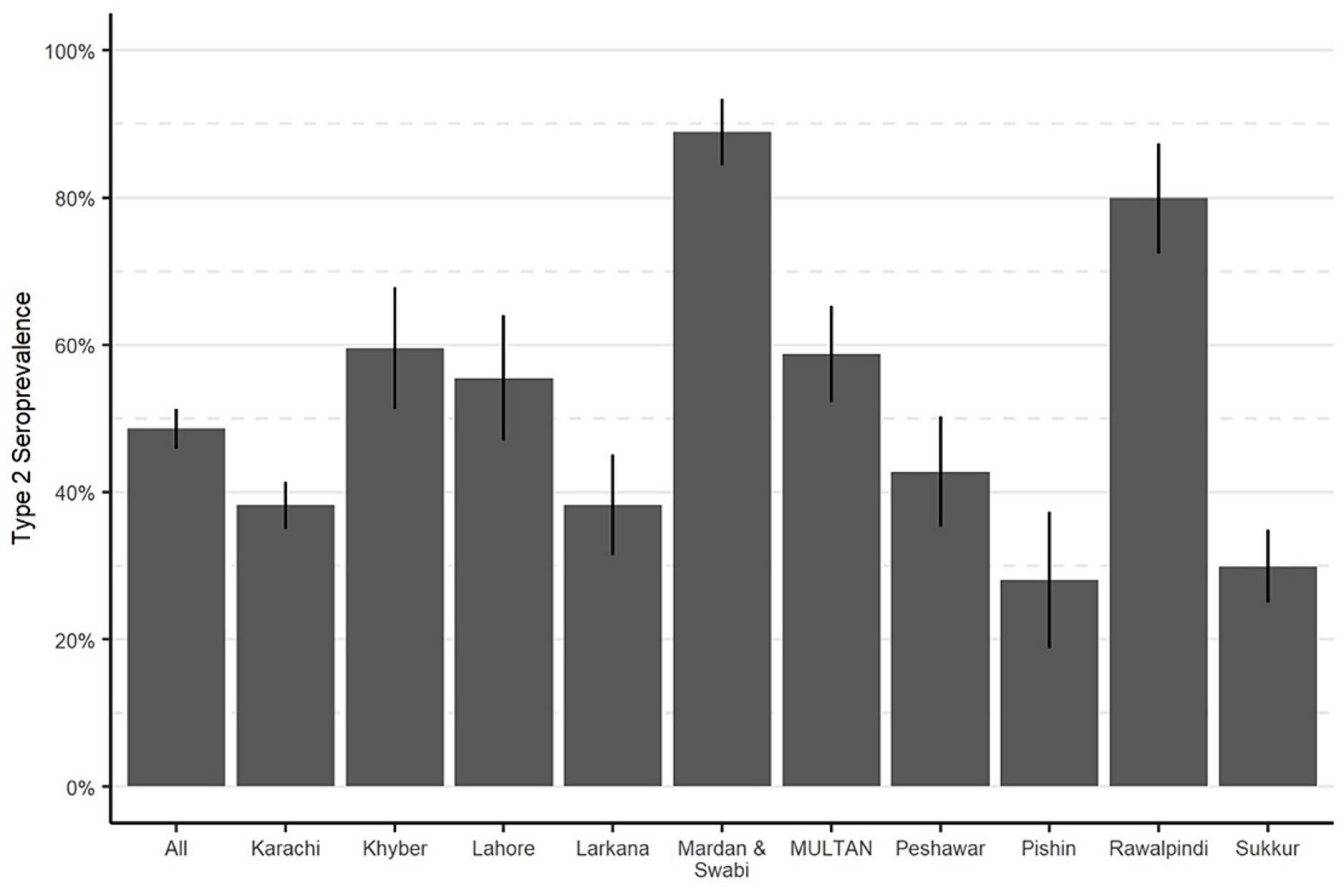
Seroprevalence of type 2 OPV in routine immunization, by study area. Bar heights represent seroprevalence, and lines show 95% confidence intervals.

To assess the effectiveness of routine immunization in protecting against type 2 poliovirus, we estimated the type 2 OPV seroprevalence among children who were born after the withdrawal of tOPV and were not eligible for mOPV2 or SIAs containing IPV. A total of 4,048 children from the study areas were born after the cessation and were not eligible for mOPV2 or IPV SIAs. Our findings indicated that among these children, the type 2 OPV seroprevalence ranged from 28% in Pishin to 89% in Mardan and Swabi. In the control sites, the OPV2 seroprevalence ranged from 55% in Lahore to 80% in Rawalpindi (Figure 4). These findings suggest that the administration of IPV through routine immunization has been effective in some areas but may require improvement in others to ensure that children receive adequate protection. For instance, IPV coverage was 33.2% in Pishin, whereas in Mardan and Swabi, it reached 91.3% (Table 1).

### Effectiveness of supplementary mOPV2 campaigns

**Figure 5:**
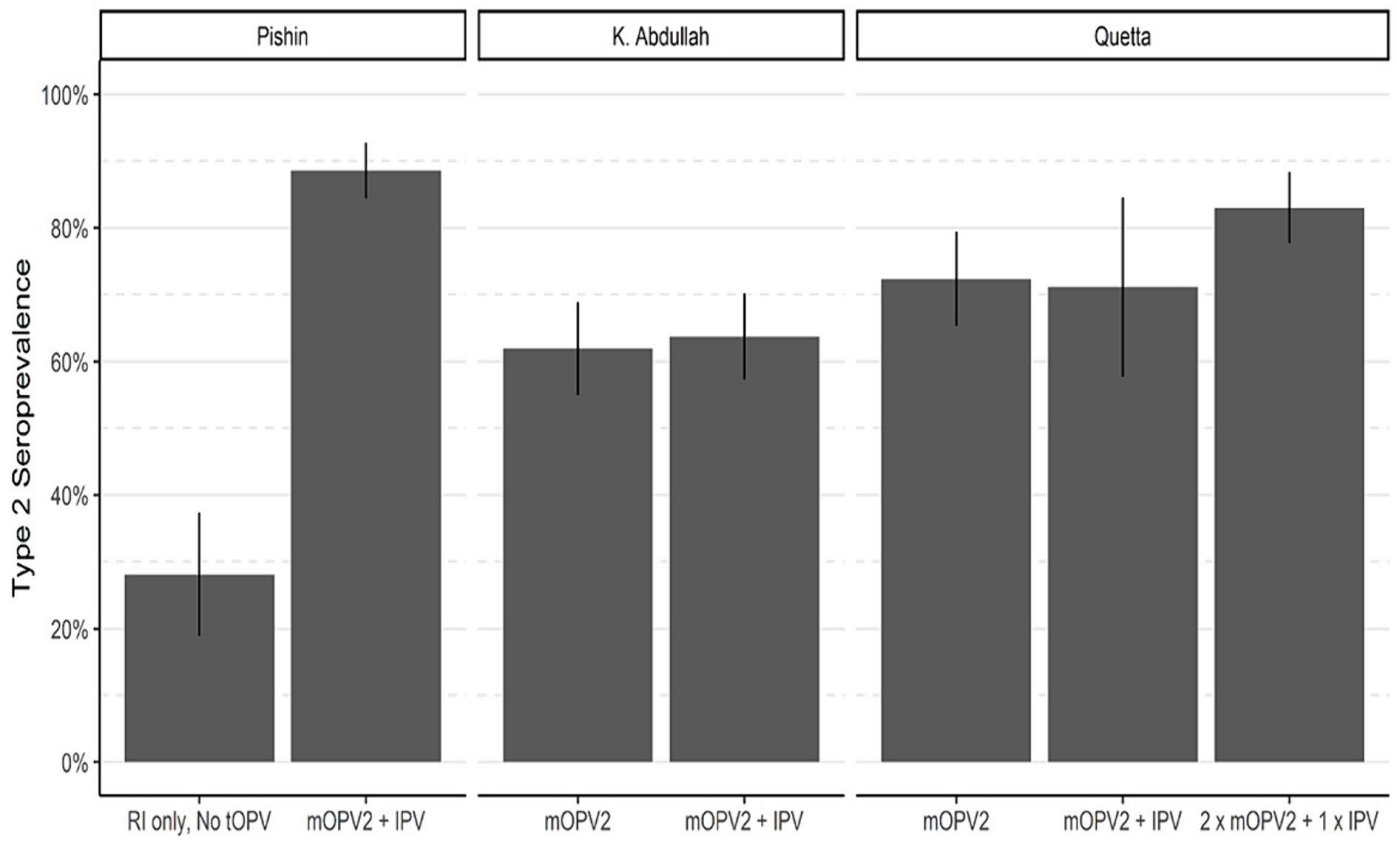
Seroprevalence of type 2 OPV in areas targeted for mOPV2 campaigns. Bar heights represent seroprevalence, and lines show 95% confidence intervals.

To evaluate the effect of mOPV2 campaigns on population immunity, the response to cVDPV2 outbreak campaigns in Baluchistan was analyzed (Figure 5). Our analysis revealed that the seroprevalence of type 2 among children in Pishin was only 28%, following the cessation of tOPV and routine immunization with IPV (Figure 5; Table 3; Supplementary Figures S1, S2). However, from August 30 to September 06, 2018, a subsequent mOPV2 + IPV SIA campaign was carried out, resulting in a significant improvement of the seroprevalence to 89% among those children who were eligible for the combined mOPV2 and SIAs with IPV in the district. The intention-to-treat (ITT) seroconversion was 85% (95% CI: 76–90%) for the combined campaign.

**Table 3:** Effectiveness of IPV and mOPV2 campaigns on type 2 seroprevalence – all survey rounds.

Area	Intervention	Reference group		Comparison group		Intention-to- treat Seroconversion (%) (95% CI)
		Vaccines	Seroprevalence (%) (95% CI)	Vaccines (in addition to reference)	Seroprevalence (%) (95% CI)	
Lahore	Routine only	Routine only	56 (47, 64)			
Rawalpindi		Routine only	80 (72, 88)			
Multan		Routine only	59 (52, 65)			
Karachi	IPV SIAs	Routine only	38 (35, 41)	IPV SIA	57 (51, 62)	29 (19, 39)
Peshawar		Routine only	43 (35, 50)	IPV SIA	76 (72, 80)	57 (48, 66)
Sukkur		Routine only	30 (25, 35)	IPV SIA	87 (82, 91)	81 (73, 87)
Larkana		Routine only	38 (31, 45)	IPV SIA	61 (52, 70)	37 (19, 52)
Khyber		Routine only	60 (51, 68)	IPV SIA	88 (83, 93)	70 (54, 81)
Mardan & Swabi		Routine only	89 (84, 93)	IPV SIA	77 (70, 84)	
Pishin	mOPV2 + IPV SIAs	Routine only	28 (18, 37)	mOPV2 + IPV SIA	89 (84, 94)	85 (76, 90)
Killa Abdullah		mOPV2 SIA	62 (54, 68)	IPV SIA	65 (53, 74)	4 (−36, 32)
Quetta		mOPV2 SIA	72 (64, 80)	mOPV2 + IPV SIA	83 (78, 88)	39 (8, 60)

CI, confidence interval; IPV, inactivated polio vaccine; SIA, supplementary immunization activities; mOPV2, monovalent oral poliovirus type 2 vaccine.

In district Killa Abdullah, the seroprevalence was 62% in the first phase of the survey which was conducted following the February 2017 mOPV2 SIA, but before to the April 2017 SIA containing IPV (Figure 5; Table 3). The third round was conducted in October 2018, and the seroprevalence among those children who were eligible for mOPV2 SIA and the additional SIA containing IPV increased to 65%. This resulted in an ITT seroconversion of 4% for the SIA containing IPV that was conducted after the SIA consisted of mOPV2 (95% CI, −36–32%) during the third round of the survey.

In district Quetta, the first round of seroprevalence testing was carried out from January 2 to 6, 2017 following a supplementary immunization activity targeted for mOPV2. The blood samples were mainly collected during the second mOPV2 SIA. 72% of the children who qualified for the mOPV2 SIA had a type 2 OPV seroprevalence (Figure 5; Table 3). For the third round of the survey in Quetta, the study included children who were either eligible for two SIAs of mOPV2 and a single SIA containing IPV, or the younger birth cohort born after January 2017 and qualifying for the second mOPV2 SIA solely and the succeeding IPV SIA. Seroprevalence for Type 2 was 71% for the younger cohort born after January 2017 who were eligible for mOPV2 + IPV and 83% for those who were eligible for 2 x mOPV2 + 1 x IPV SIAs. The resulting ITT seroconversion was 39% (95% CI: 8–60%) for the combined SIAs containing both mOPV2 and IPV. Overall, the results showed that mOPV2 campaigns combined with IPV SIA were effective in improving population immunity to poliovirus type 2 in Baluchistan.

### Effectiveness of supplementary IPV campaigns

**Figure 6:**
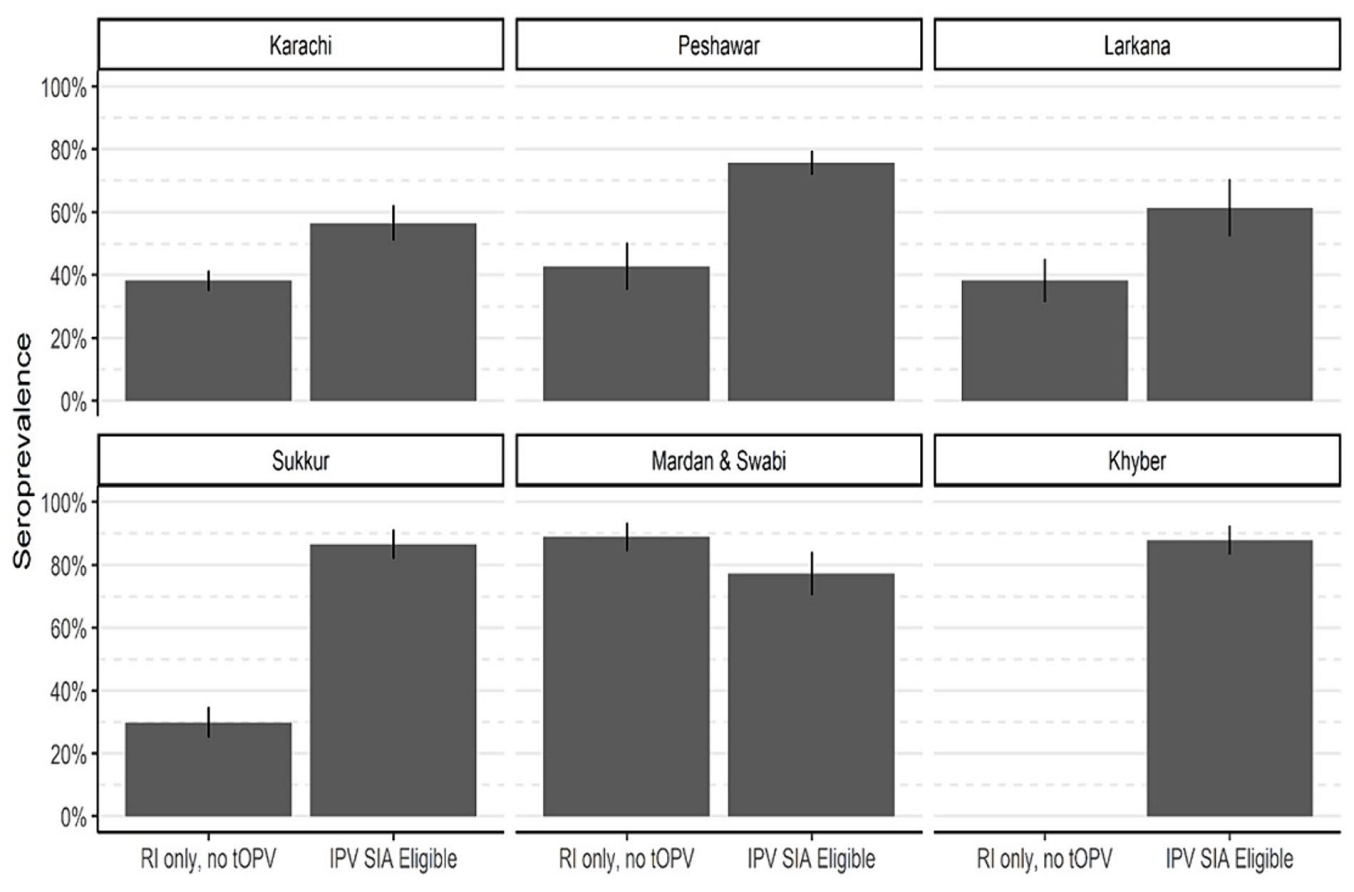
Type 2 immunity by eligibility for SIA contain IPV. The bar heights represent seroprevalence, and lines show 95% confidence intervals.

Campaigns targeting IPV took place in Karachi, Peshawar, Sukkur, Larkana, Khyber, and Mardan & Swabi in response to WPV1 outbreaks. Figure 6 and Table 3 present seroprevalence results following the IPV SIAs. The first phase of the survey in Karachi included children born after the withdrawal of tOPV and were not eligible for SIAs containing IPV. The type 2 seropositivity among those born after the withdrawal of tOPV was 38% compared to 57% among those who were eligible for the SIA containing IPV. As a result, the ITT seroconversion rate was 29% (95% CI: 19–39%).

Similarly, in Peshawar, the seropositivity increased from 43 to 76%, resulting in ITT seroconversion of 57% (95% CI: 48–66%). In district Sukkur, the IPV SIA has a significant impact on seroprevalence, increasing seropositivity from 30 to 87% with an ITT seroconversion of 81% (95% CI: 73–87%). In Larkana, the SIAs had a positive effect on immunity. The immunity rates increased from 38% among those receiving routine vaccinations in the first round of the survey to 61% among those in the succeeding survey rounds and were eligible for an SIA targeted at IPV. This increase is consistent with an ITT seroconversion of 37% (95% CI: 19–52%). In Mardan & Swabi, blood samples were not collected for reference group prior to the SIA containing IPV was conducted. However, the seroprevalence was 89%, and in the third round of the survey, it decreased to 77%. In Khyber Agency, seroprevalence increased from 60 to 88% in the third round of IPV SIA, and resulted in an ITT seroconversion rate of 70% (95 CI; 54–81%).

## Discussion

These series of surveys provide crucial insights into the seroprotection levels against type 2 poliovirus among children aged 6–11 months in Pakistan. The surveys compared findings on children born before and after the tOPV cessation, as well as before and after SIAs containing mOPV2 and several IPV campaigns that were conducted in Pakistan while responding to WPV1 and cVDPV breakout. Our study found that while immunity for type 1 and type 3 OPV was more than 90%, immunity to type 2 OPV varied across the survey areas including the control sites of the study. This variation was due to a change in vaccination regimen from tOPV to bOPV after the cessation of tOPV from routine immunization. Furthermore, there were differences in routine immunization coverage across geographical areas and birth cohorts. For children born before the cessation of tOPV from routine immunization and SIAs, the immunity to type 2 OPV was over 75%. However, for others born after the switch to bOPV, the immunity dropped to 25% and later improved to over 50% after the introduction of mOPV2.

The findings of this study are along with the findings from two prior studies carried out in Pakistan. The first study was a seroprevalence study conducted in polio high-risk districts of Pakistan, and the second study quantified immunity and field efficacy of type-2-containing polio vaccines after the withdrawal of tOPV. Both of these studies found higher prevalence of PV1 and PV3 and lower prevalence of PV2 among children aged 6–11 months (9, 24). In 2017, two similar studies were carried out in Afghanistan. One study was conducted in Kandahar province (25), and the other was a facility-based study conducted in 14 polio high-risk provinces of Afghanistan (26). Both studies found high prevalence of PV1 and PV3 in children of both age brackets (aged between 6 to 11 months and between 36 to 48 months), with low prevalence of PV2 in the younger age group compared to the older age group. Comparable findings were also documented in a study that evaluated population immunity in a continually high-risk area for WPV transmission in Western Uttar Pradesh in India (27).

We assessed the impact of SIAs utilizing mOPV2 and IPV and noticed a significant response to the combined effect of mOPV2 and SIAs that contain IPV. Following the outbreak of cVDPV2 in Baluchistan, the type 2 seropositivity rate for type 2 among 6–11 months old children in Pishin was 28% after the routine immunization. However, among children who were eligible for mOPV2 and IPV SIAs, this rate increased to 89%, resulting in overall ITT seropositivity of 85%. In Killa Abdullah, the seropositivity rate among children eligible for mOPV2 SIA was 62%, but it increased to 65% with additional IPV SIA. The incremental change in seropositivity rate for type 2 in Killa Abdullah may be explained by the shift in the vaccination regime from tOPV to bOPV, accompanied by low coverage of OPV 3 and IPV compared to other regions. In Quetta, the seropositivity with mOPV2 SIA alone was 72%, which improved to 83% with an additional SIA containing mOPV2 and IPV. These findings are consistent with other studies (9, 28), and demonstrate that mOPV2 and IPV SIAs have a substantial impact on type 2 seroprotection in a population with low immunity.

We also found that SIAs containing IPV had a significant impact on improving type 2 seroprotection among children with low immunization coverage. Seropositivity significantly increased in all areas where the campaigns were conducted. Consistent with previous studies (14, 28), these findings highlight the importance of continued efforts to implement effective vaccination strategies in areas with low immunization coverage to prevent the re-emergence of poliovirus.

Although immunity against type 2 poliovirus improved in the last survey round in 2019 due to successive SIAs, especially in Baluchistan, there is a likelihood that it declined in 2020 due to the suspension of all immunization activities during the pandemic.

In this study, the cross-sectional observation design posed a limitation as the age groups and the collection of sample were not specifically designed to assess the impact of the SIA and routine immunization. Thus, while age plays a crucial role in vaccine efficacy, the age at which an SIA conducted varies between comparison groups. Furthermore, the current study design was inadequate in explaining the differences in immunity among children across the survey areas.

## Conclusion

Immunity to type 2 OPV varied across the survey areas and birth cohorts, with a significant drop observed after the change from tOPV to bOPV. However, the implementation of mOPV2 and IPV SIAs had a significant impact in improving seroprotection against type 2 virus, especially in areas with low immunization coverage. These findings highlight the importance of implementing effective vaccination strategies to prevent the re-emergence of poliovirus. Moreover, the results provide crucial information for policymakers working toward achieving global polio eradication. Therefore, policymakers may consider continuing and possibly expanding the use of mOPV2 and IPV SIAs to maintain and further enhance population immunity against poliovirus type 2 in Pakistan. Despite the progress made, the suspension of immunization activities during the pandemic may have resulted in a decline in immunity levels, emphasizing the need for continued efforts to maintain high immunization coverage.

## Data availability statement

The original contributions presented in the study are included in the article/Supplementary material, further inquiries can be directed to the corresponding author.

## Ethics statement

The study was conducted according to the guidelines of the Declaration of Helsinki and approved by the Ethical Review Committee of Aga Khan University, Pakistan [ERC- 1658] and the National Bioethics Committee, Pakistan. The studies were conducted in accordance with the local legislation and institutional requirements. Written informed consent for participation in this study was provided by the participants’ legal guardians/next of kin.

## Author contributions

IH: Methodology, Project administration, Resources, Writing – original draft, Writing – review & editing. MU: Resources, Writing – original draft, Writing – review & editing. AK: Writing – original draft, Writing – review & editing. MS: Data curation, Formal analysis, Writing – review & editing. IA: Data curation, Formal analysis, Visualization, Writing – review & editing. KB: Resources, Writing – review & editing. JI: Resources, Writing – review & editing. MA: Writing – review & editing. RS: Writing – review & editing. SB: Writing – review & editing. AV: Writing – review & editing. JP: Writing – review & editing. SS: Conceptualization, Data curation, Funding acquisition, Investigation, Methodology, Project administration, Resources, Supervision, Validation, Visualization, Writing – review & editing.

## Supplementary material

SUPPLEMENTARY FIGURE S1 Seroprevalence in targeted areas.

SUPPLEMENTARY FIGURE S2 Type 2 neutralizing titer in areas targeted for mOPV2 campaigns.

## References

[1] Moffett DB Llewellyn A Singh H Saxentoff E Partridge J Boualam L. Progress toward poliovirus containment implementation – worldwide, 2019–2020. MMWR Morb Mortal Wkly Rep. (2020) 69:1330–3. doi: 10.15585/mmwr.mm6937a7, PMID: 32941411 PMC7498175

[2] Chard AN Datta D Tallis G Burns CC Wassilak SGF Vertefeuille JF. Progress toward polio eradication — worldwide, January 2018–march 2020. MMWR. (2020) 69:784–9. doi: 10.15585/mmwr.mm6925a4, PMID: 32584798 PMC7316320

[3] Haqqi A Zahoor S Aftab MN Tipu I Rehman Y Ahmed H. COVID-19 in Pakistan: impact on global polio eradication initiative. J Med Virol. (2020) 93:141–3. doi: 10.1002/jmv.26240, PMID: 32603503 PMC7361542

[4] Pakistan Polio Eradication Programme. (2023). Polio cases in provinces. Available at: https://www.endpolio.com.pk/polioin-pakistan/polio-cases-in-provinces

[5] Din M Ali H Khan M Waris A Ullah S Kashif M. Impact of COVID-19 on polio vaccination in Pakistan: a concise overview. Rev Med Virol. (2021) 31:e2190. doi: 10.1002/rmv.2190, PMID: 33176028

[6] Hussain I Khan A Rhoda DA Ahmed I Umer M Ansari U. Routine immunization coverage and immunization card retention in Pakistan: results from a cross-sectional National Survey. Pediatr Infect Dis J. (2023) 42:260–70. doi: 10.1097/INF.0000000000003804, PMID: 36728580 PMC9935567

[7] Platt LR Estivariz CF Sutter RW. Vaccine-associated paralytic poliomyelitis: a review of the epidemiology and estimation of the global burden. J Infect Dis. (2014) 210:S380–9. doi: 10.1093/infdis/jiu184, PMID: 25316859 PMC10424844

[8] Konopka-Anstadt JL Campagnoli R Vincent A Shaw J Wei L Wynn NT. Development of a new oral poliovirus vaccine for the eradication end game using codon deoptimization. NPJ Vaccines. (2020) 5:26. doi: 10.1038/s41541-020-0176-7, PMID: 32218998 PMC7083942

[9] Voorman A Habib MA Hussian I Safdar RM Ahmed JA Weldon WC. Immunity and field efficacy of type 2-containing polio vaccines after cessation of trivalent oral polio vaccine: a population-based serological study in Pakistan. Vaccine. (2020) 5:100067. doi: 10.1016/j.jvacx.2020.100067 PMC7240192 32462141

[10] Bandyopadhyay AS Garon J Seib K Orenstein WA. Polio vaccination: past, present and future. Future Microbiol. (2015) 10:791–808. doi: 10.2217/fmb.15.19, PMID: 25824845

[11] Pons-Salort M Molodecky NA O'Reilly KM Wadood MZ Safdar RM Etsano A. Population immunity against Serotype-2 poliomyelitis leading up to the global withdrawal of the Oral poliovirus vaccine: Spatio-temporal modelling of surveillance data. PLoS Med. (2016) 13:e1002140. doi: 10.1371/journal.pmed.1002140, PMID: 27701425 PMC5049753

[12] Garon J Seib K Orenstein WA Gonzalez AR Blanc DC Zaffran M. Polio endgame: the global switch from tOPV to bOPV. Expert Rev Vaccines. (2016) 15:693–708. doi: 10.1586/14760584.2016.1140041, PMID: 26751187

[13] GPEI. (2016) Global synchronisation and the switch. Available at: https://polioeradication.org/news-post/global-synchronisation-and-the-switch/#:~:text=What%20is%20the%20switch%3F,of%20another%20vaccine%20in%20history (Accessed March 27, 2023).

[14] Taniuchi M Famulare M Zaman K Uddin MJ Upfill-Brown AM Ahmed T. Community transmission of type 2 poliovirus after cessation of trivalent oral polio vaccine in Bangladesh: an open-label cluster-randomised trial and modelling study. Lancet Infect Dis. (2017) 17:1069–79. doi: 10.1016/S1473-3099(17)30358-4, PMID: 28693854 PMC5610141

[15] Concepción FE Mark AP Abhijeet A Steven GFW Roland WS Jay DW. Poliovirus vaccination options for achieving eradication and securing the endgame. Curr Opin Virol. (2013) 3:309–15. doi: 10.1016/j.coviro.2013.05.007, PMID: 23759252 PMC10395005

[16] Famulare M Selinger C McCarthy KA Eckhoff PA Chabot-Couture G. Assessing the stability of polio eradication after the withdrawal of oral polio vaccine. PLoS Biol. (2018) 16:e2002468. doi: 10.1371/journal.pbio.2002468, PMID: 29702638 PMC5942853

[17] Gamougam K Jeyaseelan V Jones KAV Mainou BA Palmer T Diaha A. A survey to assess serological prevalence of poliovirus antibodies in areas with high-risk for vaccine-derived poliovirus transmission in Chad. J Pediatric Infect Dis Soc. (2022) 11:55–9. doi: 10.1093/jpids/piab103, PMID: 34791366 PMC8865003

[18] Hird TR Grassly NC. Systematic review of mucosal immunity induced by Oral and inactivated poliovirus vaccines against virus shedding following Oral poliovirus challenge. PLoS Pathog. (2012) 8:e1002599. doi: 10.1371/journal.ppat.1002599, PMID: 22532797 PMC3330118

[19] Alleman MM Jorba J Greene SA Diop OM Iber J Tallis G. Update on vaccine-derived poliovirus outbreaks – worldwide, July 2019 – February 2020. MMWR. (2020) 69:489–95. doi: 10.15585/mmwr.mm6916a1, PMID: 32324719 PMC7188410

[20] Weldon WC Oberste MS MA P. Standardized methods for detection of poliovirus antibodies. New York: Springer (2016). 10.1007/978-1-4939-3292-4_8 26983734

[21] Plotkin SA. Correlates of protection induced by vaccination. Clin Vaccine Immunol. (2010) 17:1055–65. doi: 10.1128/CVI.00131-10 20463105 PMC2897268

[22] Sreevatsava M Burman AL Wahdan A Safdar RM O'Leary A Amjad R. Routine immunization coverage in Pakistan: a survey of children under 1 year of age in community-based vaccination areas. Vaccine. (2020) 38:4399–404. doi: 10.1016/j.vaccine.2020.04.068, PMID: 32402754

[23] Team RC. R: A language and environment for statistical computing. Vienna: Foundation for Statistical Computing (2015).

[24] Hussain I Mach O Habib A Bhatti Z Suhag Z Oberste MS. Seroprevalence of anti-polio antibodies in children from polio high-risk areas of Pakistan: a cross-sectional survey 2015-2016. Pediatr Infect Dis J. (2017) 36:e230–6. doi: 10.1097/INF.0000000000001622, PMID: 28806355 PMC9131303

[25] Hussain I Mach O Hamid NA Bhatti ZS Moore DD Oberste MS. Seroprevalence of anti-polio antibodies in children from polio high risk area of Afghanistan: a cross sectional survey 2017. Vaccine. (2018) 36:1921–4. doi: 10.1016/j.vaccine.2018.02.055, PMID: 29510918 PMC5873527

[26] Soofi SB Martinez M Farag NH Hendley WS Ehrhardt D Ahmed I. Poliovirus immunity among children aged 6–11 and 36–48 months in 14 polio high-risk provinces of Afghanistan: a Health-facility-based study. Vaccine. (2022) 10:1726. doi: 10.3390/vaccines10101726, PMID: 36298591 PMC9610936

[27] Deshpande JM Bahl S Sarkar BK Estívariz CF Sharma S Wolff C. Assessing population immunity in a persistently high-risk area for wild poliovirus transmission in India: a serological study in Moradabad, Western Uttar Pradesh. J Infect Dis. (2014) 210 Suppl 1:S225–33. doi: 10.1093/infdis/jiu204, PMID: 25316839 PMC10325558

[28] Voorman A Hoff NA Doshi RH Alfonso V Mukadi P Muyembe-Tamfum JJ. Polio immunity and the impact of mass immunization campaigns in the Democratic Republic of the Congo. Vaccine. (2017) 35:5693–9. doi: 10.1016/j.vaccine.2017.08.063, PMID: 28882442 PMC5628608

