# Supplementary figures and images for: Exploring the path to polio eradication: insights from consecutive seroprevalence surveys among Pakistani children
Source: Front Public Health. 2024 Mar 27;12:1384410. doi: 10.3389/fpubh.2024.1384410 (PMC11004230; doi:10.3389/fpubh.2024.1384410)

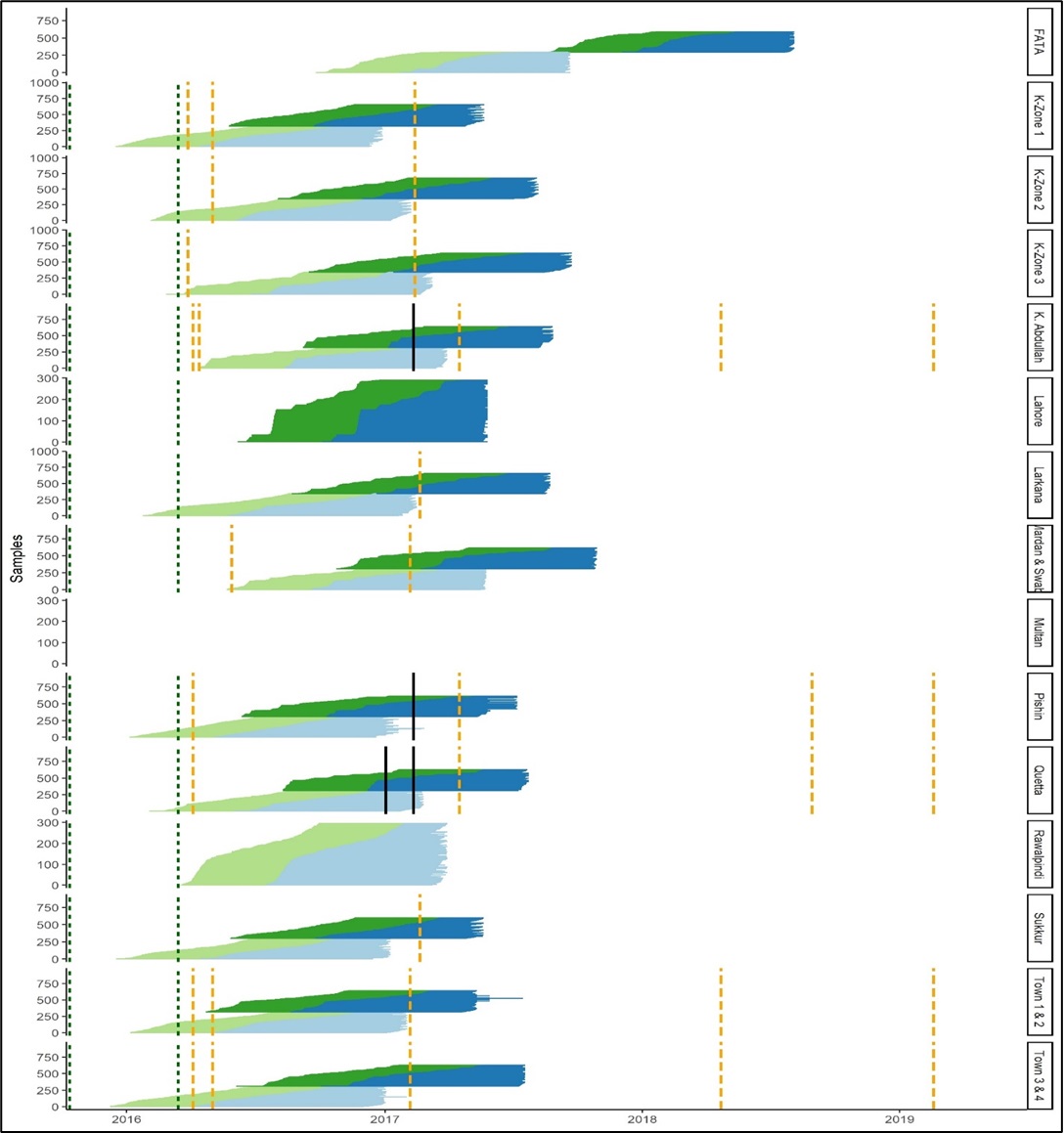

Supplement: SUPPLEMENTARY FIGURE S1 — Seroprevalence in targeted areas. [file Image_1.JPEG]

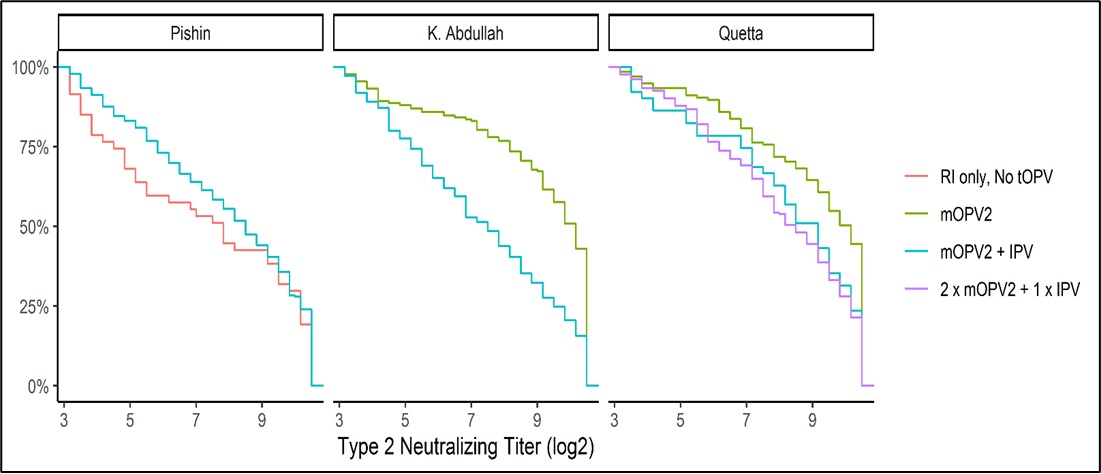

Supplement: SUPPLEMENTARY FIGURE S2 — Type 2 neutralizing titer in areas targeted for mOPV2 campaigns. [file Image_2.JPEG]
